# The Role of Oxygen Homeostasis and the HIF-1 Factor in the Development of Neurodegeneration

**DOI:** 10.3390/ijms25094581

**Published:** 2024-04-23

**Authors:** Elena V. Mitroshina, Maria V. Vedunova

**Affiliations:** Institute of Biology and Biomedicine, Lobachevsky State University of Nizhny Novgorod, 23 Gagarin Avenue, 603022 Nizhny Novgorod, Russia; mvedunova@yandex.ru

**Keywords:** hypoxia, HIF-1, neurodegeneration, neuroprotection, PHD, Alzheimer’s disease, Parkinson’s disease

## Abstract

Understanding the molecular underpinnings of neurodegeneration processes is a pressing challenge for medicine and neurobiology. Alzheimer’s disease (AD) and Parkinson’s disease (PD) represent the most prevalent forms of neurodegeneration. To date, a substantial body of experimental evidence has strongly implicated hypoxia in the pathogenesis of numerous neurological disorders, including AD, PD, and other age-related neurodegenerative conditions. Hypoxia-inducible factor (HIF) is a transcription factor that triggers a cell survival program in conditions of oxygen deprivation. The involvement of HIF-1α in neurodegenerative processes presents a complex and sometimes contradictory picture. This review aims to elucidate the current understanding of the interplay between hypoxia and the development of AD and PD, assess the involvement of HIF-1 in their pathogenesis, and summarize promising therapeutic approaches centered on modulating the activity of the HIF-1 complex.

## 1. Introduction

The aging population in most developed nations underscores the critical importance of investigating the endogenous molecular mechanisms that may mitigate or decelerate the onset of age-related diseases, particularly neurodegenerative conditions, which are of paramount concern. Neurodegenerative diseases predominantly manifest as sporadic conditions, with fewer instances of hereditary transmission, affecting the central nervous system and resulting in the gradual and progressive deterioration of specific neuronal populations and their interconnections [1]. Given the ongoing aging trend and the extension of our life expectancy, the incidence of dementia is anticipated to rise significantly in the coming years [2,3].

According to the World Health Organization (WHO), Alzheimer’s disease (AD) is the most prevalent form of dementia, accounting for an estimated 60–70% of cases globally and currently afflicting 50 million individuals worldwide [4]. Parkinson’s disease (PD) ranks as the second most common neurodegenerative disorder [5], representing the foremost serious movement disorder globally. Importantly, PD surpasses other neurological disorders in terms of escalating rates of disability and mortality. Despite advancements, effective pharmacological interventions capable of preventing or halting the progression of neurodegenerative processes in PD, AD, and other chronic neurodegenerative disorders remain elusive. Therefore, identifying the key signaling pathways and genetic mechanisms amenable to pharmacological modulation to mitigate neurodegenerative changes in the brain represents a crucial yet unresolved challenge.

To date, a substantial body of experimental evidence has strongly implicated hypoxia in the pathogenesis of numerous neurological disorders, including Alzheimer’s disease, Parkinson’s disease, and other age-related neurodegenerative conditions [6]. For instance, several studies suggest that hypoxia during the perinatal period may serve as a precipitating factor for the onset of Alzheimer’s disease later in adulthood [7]. Brain aging is linked with the emergence of chronic regions of microhypoxia within the nervous tissue. Prolonged exposure to hypoxia precipitates neuroinflammation by activating microglia, the resident immune cells in the brain. Chronic inflammation constitutes a fundamental component of neurodegenerative processes, leading to heightened levels of reactive oxygen species (ROS) and pro-inflammatory cytokines, which are hallmark features of various degenerative diseases of the central nervous system [8].

Consequently, there is a rationale for considering the intracellular systems responsible for adapting to hypoxic injury potential targets for preventing and treating neurodegenerative processes. The discovery of the transcription factor HIF-1 and its stability modulation using oxygen-dependent HIF prolyl hydroxylase started a new era in comprehending adaptive cell responses to hypoxic conditions. Greg Semenza, William Kaelin Jr., and Sir Piter Ratcliffe get the 2019 Nobel Prize in Physiology or Medicine for this research. HIF is a transcription factor that triggers a cell survival program in conditions of oxygen deprivation, activating over 100 genes that encompass various cellular processes involved in hypoxia adaptation, including angiogenesis, erythropoiesis, energy metabolism, cell proliferation, and cell cycle control [9]. Notably, the most renowned targets activated by HIF are the erythropoietin (EPO) and vascular endothelial growth factor (VEGF) genes. Specifically, the transcription factor HIF-1 can regulate the expression of numerous genes that mitigate neuronal cell death during neurodegeneration. For instance, the regulation of HIF-1 target genes involved in glycolysis or vascular regulation constitutes an early adaptive response to oxidative stress, potentially attenuating cognitive decline and delaying the progression to more severe stages of Alzheimer’s disease [10].

The involvement of HIF-1α in neurodegenerative processes presents a complex and sometimes contradictory picture. While prolonged or excessive stressors, such as ischemia or oxidative stress, may transform HIF-1 into a trigger for neuronal cell death, potentially activating the Bnip3 factor and contributing to late-onset dementia in Alzheimer’s disease [10], prolonged and severe hypoxia can induce HIF-1α-mediated stabilization of p53, subsequently triggering p53-dependent apoptosis [11]. Thus, the role of HIF-1 in the pathogenesis of neurodegenerative diseases necessitates careful examination. In this review, we initially examine the existing literature regarding the correlation between hypoxia and the onset of AD and PD. Subsequently, we delve into the role of HIF-1 in the pathophysiology of these conditions, followed by an exploration of the available data on the influence of modulating HIF-1 activity on the progression of neurodegenerative disorders.

## 2. The Role of Hypoxia in the Development of Neurodegenerative Diseases

### 2.1. The Role of Hypoxia in the Pathogenesis of Alzheimer’s Disease

**Figure 1:**
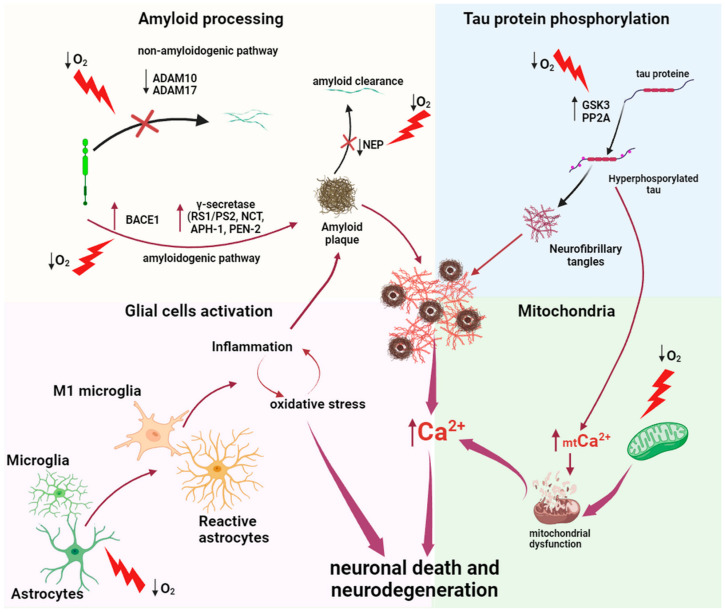
The role of hypoxia in the development of Alzheimer’s disease. Lightning indicates hypoxic exposure. Vertical up arrows indicate increasing expression or concentration, downward vertical arrows indicate decreased expression or concentration. Curved arrows show the influence of some factors on others. If the curved arrow is crossed out, this indicates an inhibitory effect.

Emerging evidence suggests that hypoxia contributes to the pathogenesis of AD through various mechanisms, including the promotion of amyloid beta (Aβ) formation, stimulation of tau hyperphosphorylation, disruption of blood–brain barrier function, and the facilitation of neuronal degeneration (Figure 1).

#### 2.1.1. Amyloid Processing

It is now known that hypoxia influences the formation and metabolism of beta-amyloid. Among the disintegrin and metalloproteinase domain-containing (ADAM) family of proteins, ADAM10 and ADAM17 are the principal amyloid precursor protein (APP)-cleaving enzymes acting at the α site of the protein [12,13]. ADAM10 predominantly functions as α-secretase in the brain, and recent identification of two rare ADAM10 mutations suggests their role as predisposing factors in early-onset AD [14]. Moreover, a single rare nonsynonymous variant rs142946965 [p.R215I] in ADAM17 was correlated with an autosomal dominant pattern of late-onset AD in one family [15]. Intriguingly, numerous studies have demonstrated that hypoxia markedly diminishes the expression and activity of the ADAM10 and ADAM17 proteins in the neurons, thereby reducing the cleavage of APP via the non-amyloidogenic pathway [16,17,18]. Under the action of α-secretases, the extracellular fragment sAPPα is generated, which exerts several neuroprotective functions, including protection against ischemic damage and glutamate neurotoxicity. Furthermore, sAPPα exhibits numerous neurotrophic properties, such as promoting neuritogenesis, synaptogenesis, and memory retention. Additionally, the sAPPα peptide inhibits the activity of beta-site APP-cleaving enzyme 1 (BACE1), thereby impeding the amyloidogenic processing of APP. Hypoxia not only suppresses α-secretase activity but also enhances the diversion of APP processing toward the amyloidogenic pathway under hypoxic conditions [19].

Research has demonstrated that chronic hypoxia upregulates the expression of β-secretase (BACE1), which facilitates amyloid precursor protein (APP) processing via the amyloidogenic pathway. The promoter region of the BACE1 gene contains a functional hypoxia-responsive element (HRE), with HIF-1α regulating BACE1 gene expression through binding to this HRE [19,20].

The γ-secretase complex, the second enzyme involved in the amyloidogenic processing pathway of APP, comprises four proteins: presenilin 1 or 2 (PS1 or PS2), nicastrin (NCT), anterior pharyngeal defect-1 (APH-1), and presenilin enhancer 2 (PEN2) [21]. Hypoxia has been found to elevate the expression of γ-secretase subunits [22]. Chronic hypoxia decreases the expression of DNA methyltransferase 3b (DNMT3β), leading to demethylation of the PEN2 CpG site and the promoters of the NCT and PS1 genes. Consequently, this results in transcriptional activation and increased protein levels of PEN2, NCT, and PS1, thereby enhancing γ-secretase activity. The promoter of the APH-1A gene contains HIF-1α-binding sites, leading to increased APH-1 expression under hypoxic conditions [23]. Therefore, studies suggest that hypoxia regulates the expression of γ-secretase complex subunits and subsequently augments its activity and amyloid beta production through epigenetic mechanisms [19,20,24].

Hypoxia diminishes the expression of Aβ-degrading enzymes, particularly the metalloprotease neprilysin (NEP), and involves the hypoxic induction of caspases [25]. The APP intracellular domain (AICD), a product of the amyloidogenic pathway, has been observed to bind to the NEP gene promoter, thereby activating its transcription. This mechanism potentially serves as a protective mechanism aimed at mitigating the neurotoxic effects of Aβ. However, AICD is also a caspase substrate. Consequently, the hypoxic induction of caspases can increase AICD cleavage, resulting in decreased NEP levels and amyloid accumulation [25].

Hypoxia can disrupt calcium homeostasis through the accumulation of Aβ, and subsequently, elevated levels of intracellular calcium can trigger cell death [26]. Ca^2+^ play a pivotal role in regulating various neuronal and astrocytic functions, including neurotransmitter release, synaptic plasticity, membrane excitability, gene transcription, cell proliferation, and cell death. The depolarization of senescent neurons induces an influx of Ca^2+^ from the extracellular space, leading to excitotoxicity [27] Elevated Ca^2+^ levels result in glutamate accumulation in the synaptic cleft, promoting the activation of postsynaptic AMPA/NMDA receptors, which further increases the neuronal calcium influx. Heightened cytosolic Ca^2+^ levels may facilitate Aβ production and its subsequent neurotoxic effects, while Aβ accumulation stimulates neuronal Ca^2+^ signaling. Consequently, a synergistic interplay between Ca^2+^ and Aβ may exacerbate neurodegeneration and cognitive impairment in individuals with Alzheimer’s disease [19,28]. 

#### 2.1.2. Mitochondria

Moreover, Aβ causes both cytosolic and mitochondrial Ca^2+^ overload both in vitro and in vivo. Misfolded and hyperphosphorylated tau protein also disrupts Ca^2+^ homeostasis in the mitochondria. This leads to mitochondrial dysfunction, the generation of ROS, and the activation of apoptosis (Figure 1). Mitochondrial dysfunction in AD is an important pathogenesis pathway and includes mitochondrial oxidative stress, disruption of cellular bioenergetics, and the induction of neuronal apoptosis [29].

However, it is important to note that although hypoxia promotes β-amyloid production, amyloidosis does not necessarily equate to the development of dementia or AD. The data on the β-amyloid content in the brain tissue of elderly individuals and centenarians vary considerably across different studies. For instance, in a study by Itoh et al., the density of senile amyloid plaques and neurofibrillary tangles in centenarians aged 101.5 ± 1.5 years was notably lower than in patients with AD (80.8 ± 3 years) but higher than in individuals without dementia symptoms at the age of 79.8 ± 3.2 years [30]. Similarly, in research conducted by Hauw et al., neuropathological examination of the brains of 20 centenarians over 100 years of age revealed persistent neurofibrillary tangles and diffuse Aβ deposits in 19 of the centenarians, with senile plaques being widespread. However, only five of them were diagnosed with dementia, including AD [31].

#### 2.1.3. Tau Protein Hyperphosphorylation

Hypoxia contributes to tau protein hyperphosphorylation by disrupting the balance between kinases and phosphatases, notably glycogen synthase kinase-3β (GSK-3β), cyclin-dependent-like kinase-5 (CDK5), and protein phosphatase 2A (PP2A). Chronic hypoxia activates protein kinases like GSK-3β and CDK5 while inhibiting phosphatase PP2A, resulting in elevated tau phosphorylation levels [32,33].

#### 2.1.4. Neuroinflammation

Neuroinflammation is a prominent consequence of cerebral hypoxia and plays a significant role in AD pathogenesis. Microglia, the resident macrophages of the central nervous system (CNS), are pivotal in mediating neuroinflammation. Hypoxia induces microglial overactivation, leading to the secretion of pro-inflammatory cytokines such as TNF-α, IL-1β, and interferon-γ. These cytokines can downregulate Aβ receptor expression and clearance while activating BACE1, ultimately causing neuronal damage and degeneration [34,35]. Furthermore, activated microglia trigger astrocyte activation, further amplifying the production of pro-inflammatory factors [36]. Furthermore, in addition to the activation of the microglia along the M1 inflammatory profile, hypoxia/ischemia likely upregulates the microglial expression of CD33 and its downstream target SHP-1, a tyrosine phosphatase that inhibits TREM2-driven phagocytosis. Consequently, the microglia fail to effectively phagocytose amyloid conglomerates, leading to the accumulation of plaques in the brain tissue. Additionally, hypoxia/ischemia promotes the deposition of sialylated gangliosides, including GM1, GM2, GM3, and GD1, which serve as ligands for inhibitory receptors such as CD33/Siglec-3 [20,37].

#### 2.1.5. Sleep Apnea as a Risk Factor in the Development of Alzheimer’s Diseases

Obstructive sleep apnea (OSA) syndrome is characterized by recurrent episodes of upper airway obstruction during sleep, leading to repetitive awakenings and episodes of hypoxia. 

There is a growing body of research suggesting a correlation between sleep apnea, hypoxia, and the onset of Alzheimer’s disease. Studies have reported that over 70% of individuals with dementia exhibit sleep-disordered breathing [38,39,40]. However, the precise mechanisms underlying how sleep disturbances, including OSA, may impact cognitive decline remain incompletely understood.

Recently, the glymphatic system of the brain was described, consisting of morphofunctional components such as Virchow–Robin spaces, spaces between the basement membrane, pericytes, astrocyte feet, the aquaporin receptor system of astrocytes, structures responsible for producing and resorbing cerebrospinal fluid, the interstitial space of the brain, and the space of liquor circulation. This system facilitates the removal of harmful substances and toxins, including Aβ42, accumulated during wakefulness from the brain. It has been demonstrated that this function is active only during sleep, particularly during slow-wave sleep. This may represent an additional mechanism by which sleep fragmentation, characteristic of OSA, may contribute to cognitive decline and the pathogenesis of Alzheimer’s disease [41,42,43].

In light of these findings, a study involving 74 boys aged 15–17 years was conducted. Significantly elevated levels of the specific protein Aβ42, a key player in cognitive impairment, were observed in the plasma of adolescents with obstructive sleep apnea syndrome (OSAS) compared to their peers without OSAS [44]. It has been proposed that the disruption of the interaction between the cerebrospinal fluid and interstitial fluid in OSAS may contribute to this. The glymphatic system, responsible for exchanging solutes between these fluids, relies on the pressure cycles associated with respiration to facilitate flow from the interstitial fluid into the cerebrospinal fluid. During episodes of obstructive apnea, elevated intrathoracic and intracranial pressure occurs due to increased breathing efforts against a closed airway, followed by a sudden pressure reversal at the end of the apnea. Repeated fluctuations in pressure may disrupt the glymphatic flow, leading to the retention of metabolites such as Aβ and other neuron-derived substances in the interstitial fluid rather than their transport into the cerebrospinal fluid [45].

Neuroinflammation is a key component observed in both neurodegenerative diseases and sleep apnea. Intermittent hypoxia, characteristic of sleep-disordered breathing, also induces microglial activation and neuroinflammation. Accumulating experimental evidence suggests that chronic neuroinflammation resulting from sleep apnea may serve as a potential mechanism underlying an increased risk of Alzheimer’s disease [46,47].

Intermittent hypoxia (IH) constitutes a significant component of obstructive sleep apnea, characterized by recurrent episodes of reduced blood oxygen levels [48].

In vivo studies have demonstrated that IH disrupts spatial learning and memory in rodents [49] while also adversely affecting hippocampal synaptic plasticity [50,51], likely mediated by heightened ROS production [52]. Increased expression of the HIF-1α protein has been observed in hippocampal neurons exposed to IH [53,54], with evidence suggesting a twofold rise in ROS expression and the impairment of N-methyl-D-aspartate receptor (NMDAr)-dependent long-term potentiation (LTP). Conversely, such effects were absent in heterozygous HIF-1α knockout animals [53].

Furthermore, IH induces increased amyloidogenic processing of APP and elevated production of beta-amyloid peptides. Short-term exposure to chronic IH for three days has been shown to significantly enhance beta-amyloid peptide production and the expression of BACE, presenilin, and HIF-1 [55].

Notably, studies indicate that modeling intermittent hypoxia in mice for 5 or 28 days induces alterations in genome methylation akin to aging, particularly affecting the metabolic cascades associated with neurogenesis, the cell cycle, and energy production, alongside an increase in phosphorylated tau protein [56].

However, some investigations have not observed elevated HIF-1 levels during IH development. For instance, in experiments modeling CIH over a prolonged period of four weeks in transgenic AD mice, it was noted that CIH significantly raised the Aβ42 levels in mice’s brains without concurrent upregulation of HIF-1α expression [57]. There were no discernible differences in the levels of the BDNF, proBDNF, and HIF-1α proteins in the peripheral blood between the OSA patient group (n = 20) and the control group (n = 20), neither in the evening before bedtime nor in the morning after sleep [58].

This observation underscores the notion that the effects of HIF-1 depend on the duration and intensity of hypoxic exposure.

Hypoxia exerts multifaceted effects on various pathogenetic aspects, including autophagy, oxidative stress, mitochondrial and synaptic dysfunction, and endoplasmic reticulum stress, all of which can contribute directly or indirectly to neurodegeneration [34].

In summary, hypoxia can trigger several mechanisms implicated in the pathogenesis of Alzheimer’s disease. Oxygen deprivation can induce the amyloidogenic cleavage of APP, leading to the accumulation of Aβ, which serves as the primary pathological trigger of AD. The pathological effects of Aβ on calcium homeostasis in the neurons and astrocytes contribute to neuronal cell death. Moreover, hypoxia not only promotes the formation and accumulation of Aβ but also independently disrupts the regulation of calcium homeostasis (in both the neurons and astrocytes) and induces oxidative stress, both of which contribute to neuronal cell death. Furthermore, Aβ accumulations may activate the microglia through a neurotoxic pathway, initiating a maladaptive neuroinflammatory response that exacerbates neurodegeneration. Additionally, hypoxia itself triggers neuroinflammation and neurodegeneration, which can be regarded as another initiating pathological trigger of AD.

### 2.2. The Influence of Hypoxia on the Development of Parkinson’s Disease

Despite accumulating evidence suggesting the potential involvement of hypoxia and/or altered responses to hypoxia in the pathogenesis of Parkinson’s disease, the precise nature of these relationships remains incompletely understood. 

Several studies propose that hypoxia may serve as a trigger for the phosphorylation and aggregation of α-synucleins. Various post-translational modifications of α-synuclein are known, including phosphorylation, ubiquitination, glycosylation, and acetylation. Among these modifications, phosphorylation at the Ser129 site is considered a key event that promotes α-synuclein aggregation and subsequent pathology [59,60]. Phosphorylated α-synuclein (p-α-syn) represents the predominant pathological form of α-synuclein, with autopsy findings from PD patients indicating that 90% of the protein composition within Lewy bodies consists of p-α-syn. Under normal physiological conditions, α-synuclein primarily exists as a disordered monomer, with minimal modifications or aggregation observed in healthy individuals. However, p-α-syn facilitates the aggregation of α-synuclein into various toxic forms, including monomers, dimers, oligomers, fibrils, and fibrous aggregates, with α-synuclein oligomers considered particularly neurotoxic [61,62]. The accumulation of misfolded and aggregated forms of α-synuclein is observed to increase under hypoxic conditions [63]. Chronic short-term hypoxia has been shown to induce the conversion of α-synuclein into p-α-syn in mouse brain models, with p-α-syn aggregation occurring more rapidly and readily compared to α-syn aggregation, suggesting a potential mechanism for the propagation of α-synuclein pathology in PD. Hypoxia has also been found to promote the formation and spread of α-synuclein pathology in mouse models, accelerating the loss of dopaminergic neurons and the onset of behavioral abnormalities [64,65]. Additionally, rodent models of hypoxia, including those induced by middle cerebral artery occlusion (MCAO) and systemic hypoxia, have demonstrated increased expression of both total α-synuclein and p-α-syn [66,67,68]. Similar findings have been observed in nerve cell cultures. In an in vitro model, the total quantity of α-syn and p-α-syn and their oligomers demonstrated an increase after subjecting cells to a medium with oxygen levels of 0.5% for 24 h or 1% for 48 h [69]. 

Recent studies have also implicated OSA as a risk factor in Parkinson’s disease. Patients with OSA exhibit significantly higher plasma levels of total α-synuclein and phosphorylated α-synuclein, which are positively correlated with disease severity and hypoxia levels. These findings suggest a potential involvement of hypoxia in Parkinson’s disease pathogenesis [70]. Furthermore, some studies indicate that OSA is associated with an increased severity of cognitive dysfunction and motor symptoms in Parkinson’s disease [71].

The association between Parkinson’s disease and hypoxia may stem from the heightened susceptibility of the dopaminergic neurons located in the substantia nigra to oxygen deprivation. These neurons exhibit an elevated rate of energy metabolism compared to those in other regions and possess a basal respiration rate approximately threefold higher. Furthermore, they manifest increased levels of glycolysis, enhanced axonal branching, a higher mitochondrial density, and heightened vulnerability to cytotoxins [72]. These factors suggest that the dopaminergic neurons in the substantia nigra are particularly vulnerable and exhibit a degree of susceptibility to hypoxia [65].

**Figure 2:**
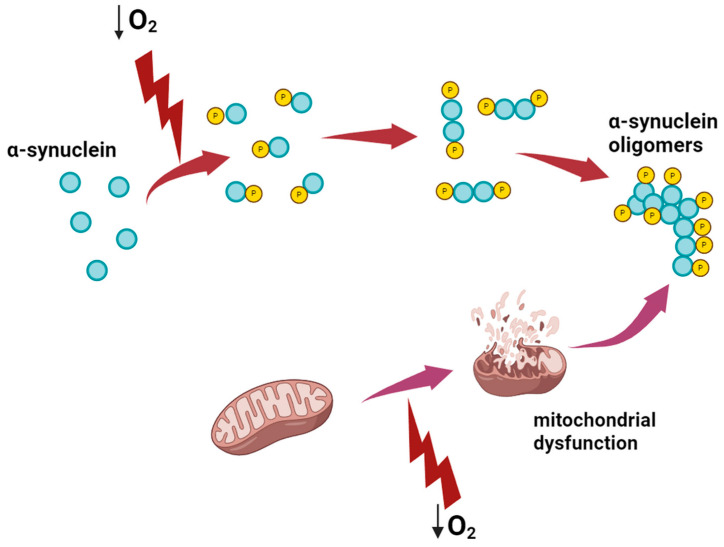
The role of hypoxia in α-syn aggregation and development of Parkinson’s disease. Lightning indicates hypoxic exposure.

Hypoxia-induced mitochondrial dysfunction may intertwine with α-syn pathology, and they may reciprocally influence each other (Figure 2). Anomalies in α-syn are believed to impact various mitochondrial functions, including the tricarboxylic acid cycle, oxidative phosphorylation, ROS and calcium homeostasis, and membrane potential. Mitochondrial dysfunction may arise from pathological interactions between α-syn and their membranes. Both soluble and aggregated forms of α-syn have been reported to engage with mitochondrial proteins, primarily facilitated by the C terminus of α-syn [73].

Multiple mitochondrial factors may influence the formation of alpha-synuclein pathology. The accumulation of ROS is associated with an increased loss of dopaminergic neurons in the substantia nigra in Parkinson’s disease. Patients with PD demonstrate a widespread reduction in antioxidant defenses in the substantia nigra pars compacta, along with decreased levels of antioxidants in the peripheral blood [74]. Hypoxia can elevate mitochondrial ROS production primarily by limiting the oxygen availability to complex III and by modulating the expression of antioxidant genes [75].

The debate persists regarding whether α-syn pathology arises from mitochondrial dysfunction or whether mitochondrial damage is a consequence of α-syn aggregation and/or pathology formation. It is proposed that other factors, particularly hypoxia and the body’s response to it, play a pivotal role in the onset of pathological processes affecting both α-syn and mitochondrial function in PD pathogenesis [73].

Furthermore, over 20 genetic mutations have been linked to PD, some of which are associated with the response to hypoxia. For instance, PARK1 (α-synuclein) is abundantly expressed in the neuronal cells, particularly at the presynaptic terminals, where it facilitates synaptic transport and neurotransmitter release. Mutations in this gene can lead to the formation of Lewy bodies, a process that may be exacerbated by hypoxia, ultimately contributing to neuronal dysfunction and death. ATP13A2 (PARK9) is a P-type ATPase involved in intracellular vesicle localization and cation homeostasis regulation. Disruptions in ATP13A2 activity due to mutations result in the development of an autosomal recessive form of PD. Given that the ATP13A2 gene promoter harbors an HRE, the stabilization of HIF-1α triggers ATP13A2 expression in dopaminergic neurons [64].

The available evidence strongly supports the notion that hypoxia represents a significant trigger for inducing neurodegenerative alterations. The primary mechanism facilitating the cellular adaptation of the brain to hypoxia is HIF-1. Consequently, the involvement of the HIF-1 factor in the pathogenesis of neurodegenerative diseases holds significant interest for researchers. Subsequently, we will explore the existing knowledge regarding the role of HIF-1 in the pathogenesis of AD and PD.

## 3. Hypoxia-Inducible Factor (HIF) in Neurodegenerative Diseases

### 3.1. Structure and Functions of HIF

The HIF family comprises heterodimeric transcription factors consisting of α and β subunits. The β subunit, also known as ARNT (aryl hydrocarbon receptor nuclear translocator), is constitutively expressed and resides within the cell nucleus continuously. In contrast, stabilization of the α subunit is governed by oxygen levels [76,77]. HIF-α encompasses three known isoforms: HIF-1α, HIF-2α, and HIF-3α (with its function still not fully elucidated). The expression patterns of HIF-1α and HIF-2α vary: HIF-1α is ubiquitously expressed in all organs, while HIF-2α expression is confined to specific tissues, such as the endothelium, kidneys, pancreas, liver, heart, lungs, intestine, and brain [78,79].

The protein structure of all HIF-α and ARNT isoforms exhibits similarity, featuring a bHLH (helix–loop–helix) domain facilitating binding to the HREs on DNA, and a PAS domain mediating subunit dimerization. HIF-α possesses a distinct oxygen-dependent degradation domain (ODDD), responsible for regulating subunit stability in an oxygen-dependent manner, along with two transactivation domains, namely the N-terminal (N-TAD) and the C-terminal (C-TAD) (excluding HIF-3α). The C-TAD domain is accountable for interactions with coactivators such as p300 and CBP [79,80].

HIF-1α accumulation occurs in response to hypoxia and undergoes rapid degradation upon reoxygenation. Under normoxic conditions, HIF-1α undergoes hydroxylation at the Pro402 and Pro564 residues within the ODDD, facilitated by prolyl hydroxylase (PHD) enzymes [77,81,82]. This hydroxylation process necessitates molecular oxygen (O_2_), iron in its ferrous state (Fe^2+^), and 2-oxoglutarate as cofactors. In its hydroxylated form, HIF-1α is recognized by the von Hippel–Lindau protein (pVHL) and E3 ubiquitin ligase, leading to its targeting for rapid ubiquitin-mediated proteasomal degradation by the 26S proteasome [83,84]. A reduced flux of oxygen induces PHD hydroxylation, disrupting the interaction between HIF-1α and pVHL, resulting in the accumulation of HIF-1α. Subsequently, the accumulated HIF-1α translocates to the nucleus and forms a complex with HIF-1β to assemble transcriptionally active HIF-1 [85]. Under hypoxic conditions, HIF-1 functions as a transcription factor primarily by associating with the HREs in gene promoters, typically characterized by the 5′-(A/G) CGTG-3′ consensus sequence. This interaction with transcriptional coactivators such as CBP and p300 modulates the expression of various genes [86]. However, in the presence of oxygen, the HIF-1 inhibitory factor (FIH) hydroxylates the asparagine residues within the C-TAD of HIF-1α, blocking its interaction with coactivators and suppressing HIF-1 transcriptional activity. Although both FIH and PHD are deactivated by hypoxia, their activity can be inhibited by iron chelators, even during healthy development, as both enzymes harbor Fe^2+^ in their catalytic sites [75].

The induction of HIF-1α and the subsequent expression of its target genes activate a pivotal signaling pathway implicated in molecular and cellular adaptation to hypoxia. HIF-1α governs the expression of a diverse array of genes involved in vasomotor control, angiogenesis, erythropoiesis, iron metabolism, cell cycle regulation, cell proliferation and death, and energy metabolism. Notably, HIF-1α facilitates the efficient transport and delivery of nutrients and oxygen by upregulating the expression of erythropoietin (EPO), which stimulates red blood cell production, and vascular endothelial growth factor A (VEGFA), which fosters angiogenesis [79,86]. Additionally, HIF orchestrates cellular metabolic adjustments to hypoxia by favoring a shift from oxidative to glycolytic metabolism. Hypoxia profoundly impacts mitochondrial energy production, as reduced oxygen availability hinders ATP synthesis in the electron transport chain [75]. Consequently, HIF-1α suppresses aerobic oxidation and promotes glycolytic metabolism [87]. This is achieved by upregulating the expression of enzymes such as hexokinase (HK) and enolase 1 (ENO1), which accelerate pyruvate production, subsequently converted into lactate by HIF-1α-induced lactate dehydrogenase (LDHA) [88]. Furthermore, HIF-1α activates the gene encoding pyruvate dehydrogenase kinase (PDK1), which inactivates the TCA cycle enzyme pyruvate dehydrogenase (PDH), which converts pyruvate into acetyl-CoA [89]. As a result, the tricarboxylic acid cycle decelerates, and the production of the electron transport chain (ETC) substrates NADH and FADH2 is attenuated. To mitigate ETC activity and reduce ROS generation, HIF-1α induces the expression of NDUFA4L2, which dampens complex I activity. Moreover, the genes that regulate cytochrome c oxidase (the ETC Complex IV that reduces O_2_ into H_2_O) are also regulated by HIFs, including the COX4-2 isoform and the LON protease, which produces a switch from isoform COX4-1 to COX4-2 that optimizes respiration efficiency under hypoxic conditions [90]. Thus, HIF orchestrates metabolic adaptation to hypoxic environments.

Genes implicated in glucose transport or glycolysis are activated by HIF, including glucose transporter-1 and 3 (GLUT 1 and 3), pyruvate dehydrogenase kinase 1 (PDK1), and lactate dehydrogenase A (LDHA). In summary, HIF-1α-mediated gene expression enhances the protection of viable cells and facilitates the removal of cells damaged by hypoxia [75,86].

### 3.2. HIF for Alzheimer’s Disease

The role of the HIF-1 factor in AD pathogenesis is dual. On the one hand, several studies indicate a decrease in HIF-1 expression in AD, likely contributing to disturbances in glucose metabolism. The evidence suggests impaired glucose uptake and metabolism in the brains of AD patients, which appear to be causes rather than consequences of neurodegeneration in AD [91]. The neurons in the brain lack the ability to synthesize or store glucose, relying entirely on the glucose transport across the blood–brain barrier facilitated by GLUT transporters, with GLUT1 and GLUT3 being predominant in the mammalian brain. GLUT1 is tasked with transporting glucose from the bloodstream into the extracellular space of the brain, while GLUT3 facilitates the transport of glucose from the extracellular space into the neurons. Reduced expression of GLUT1 and GLUT3 has been observed in AD brains [92,93,94], likely resulting from decreased levels of HIF-1α. The current data on the HIF-1 expression levels in AD patients are limited and conflicting. For instance, the brain levels of HIF-1 have been reported to be diminished in AD patients compared to age-matched healthy controls [95]. It is conceivable that reduced levels of HIF-1 in the brain are associated with increased oxidative stress, which is heightened in AD and may destabilize HIF-1 [96]. The induction of HIF-1 expression has been shown to increase GLUT1 expression, enhance glycolysis, and reduce oxidative stress [95,97]. These observations were corroborated in a study demonstrating that α-lipoic acid upregulates HIF-1α expression and restores glucose metabolism by stimulating the brain-derived neurotrophic factor (BDNF)/tropomyosin-linked kinase B (TrkB) pathway [98].

Impaired glucose uptake and metabolism have been shown to result in decreased levels of O-linked N-acetylglucosamine (O-GlcNAc) and subsequent hyperphosphorylation of tau protein, leading to the formation of neurofibrillary tangles and neuronal degeneration in the brains of AD patients. Under normal circumstances, O-glycosylation occurs, wherein β-N-acetylglucosamine (GlcNAc) attaches to the hydroxyl group of serine or threonine residues, regulating protein phosphorylation [96,99,100]. Additionally, elevated levels of HIF-1 have been reported to decrease tau protein phosphorylation and neuron inflammation, thereby offering cellular protection against apoptosis [101]. 

The levels of GLUT2, responsible for transporting glucose into the astrocytes, were found to be more than twice as high in AD brains compared to controls. Given that GLUT2 expression is mainly linked to the astrocytes in the brain, this observed increase in GLUT2 levels likely stems from astrocyte activation in AD [94]. Aβ-dependent activation of the astrocytes has been demonstrated to induce a prolonged reduction in HIF-1α expression and a decline in glycolytic rates. However, maintaining HIF-1α levels under conditions that prevent its proteasomal degradation can reverse glial activation and glycolytic alterations [102].

Several studies have highlighted the neuroprotective role of HIF-1 against beta-amyloid peptide toxicity [103,104], a topic that will be extensively discussed in subsequent sections. Therefore, HIF-1 contributes to neuronal survival during neurodegenerative processes.

An essential aspect concerning the involvement of HIF-1 in pathogenesis pertains to its impact on inflammatory mechanisms. In AD, the production of inflammatory cytokines such as TNF-α and IL-1β may activate HIF-1α, possibly by inhibiting PHD enzymes and enhancing HIF-1 stability in an oxygen-independent manner [105,106]. Subsequently, HIF-1 suppresses the expression of inflammatory cytokine receptors within the hippocampus, thereby mitigating the detrimental effects of excessive neuroinflammation [10,107]. Moreover, HIF-1α may diminish the production of pro-inflammatory cytokines like IL-6 and TNF-α by upregulating the expression of IL-10, which suppresses NLRP3 expression, thus modulating inflammasome activation and caspase-8 activation [108]. Alternatively, HIF-1α may exert its anti-inflammatory effects through the IL-10/JAK1/STAT3-mediated signaling pathway [109].

Contrary evidence suggests that HIF-1 may stimulate the production of inflammatory cytokines such as TNF-α, leading to the establishment of a positive feedback loop between hypoxia and inflammation [110]. Recent investigations by Jung et al. [111] on transgenic 3xTg AD mice revealed a reduction in capillary diameter alongside heightened expression of HIF-1 in the cerebral vascular endothelium. The authors proposed that HIF-1 in the vascular endothelial cells triggers the upregulation of the NADPH oxidase Nox4, instigating an inflammatory response. Additionally, the involvement of the NLRP1 inflammasome in this cascade has been underscored [112]. This line of inquiry aligns with the findings of P. Grammas, who demonstrated increased HIF-1α immunoreactivity in brain tissue from Tg (APPSWE) 2576-AD mice aged 50 to 60 weeks compared to Tg 2576 controls [112].

Further insights underscore that HIF-1 induces an augmentation in the expression of BACE1 and γ-secretase, pivotal factors in the amyloidogenic cleavage of APP, thereby indicating a direct positive association between HIF-1 and AD pathogenesis [113]. Moreover, HIF-1-deficient mice exhibit diminished BACE1 expression in select brain regions, including the hippocampus and cerebral cortex, highlighting the significant role of hypoxia/HIF-1 in modulating amyloidogenic APP processing. Guglielmotto et al. posited that reactive oxygen species (ROS) primarily drive the early activation of BACE1 following hypoxia, with subsequent activation of HIF-1α exacerbating BACE1 activity in later stages [114].

Moreover, cerebral ischemia and stroke have been shown to upregulate HIF-1α expression, which, via a pro-apoptotic pathway involving p53, augments the expression of BACE1, thereby contributing to the amyloidogenic process. While moderate hypoxia elicits the protective effects of HIF-1, severe hypoxia can lead to elevated levels of HIF-1α, activating the tumor suppressor gene p53 and subsequently inducing cellular apoptosis [103,115].

In work [116] observed that hypoxia induces cell death and autophagy in the microglia by elevating HIF-1α expression. In vitro studies revealed that the suppression of HIF-1α using pharmacological inhibitors (3-MA, Baf A1) or RNA interference mitigated microglial death and autophagy [116]. It is plausible that HIF-1α exacerbates the secretion of proinflammatory factors by the microglia while inhibiting phagocytosis.

Furthermore, the involvement of the HIF-1 factor in AD pathogenesis is intertwined with iron metabolism in brain tissue. Elevated iron levels may heighten toxic Aβ deposition and the hyperphosphorylation of tau protein [117,118]. A key regulator of the iron content and distribution in the brain is the TfR1 protein. Increasing evidence suggests that TfR gene transcription is modulated by hypoxia, with HIF-1α (the best studied transcriptional activator of hypoxia-responsive genes) playing a pivotal role in this regulation [119,120]. Recent investigations have demonstrated the upregulation of TfR1 protein expression in the cerebral cortex of 8-month-old female 5xFAD mice [121], which is associated with the activation of the HIF-1 signaling pathway, oxidative stress, and inflammation. Remarkably, this study revealed elevated HIF-1α mRNA expression in the cortical tissues of 5xFAD mice, alongside stabilization and nuclear translocation of the HIF-1 complex [121].

**Figure 3:**
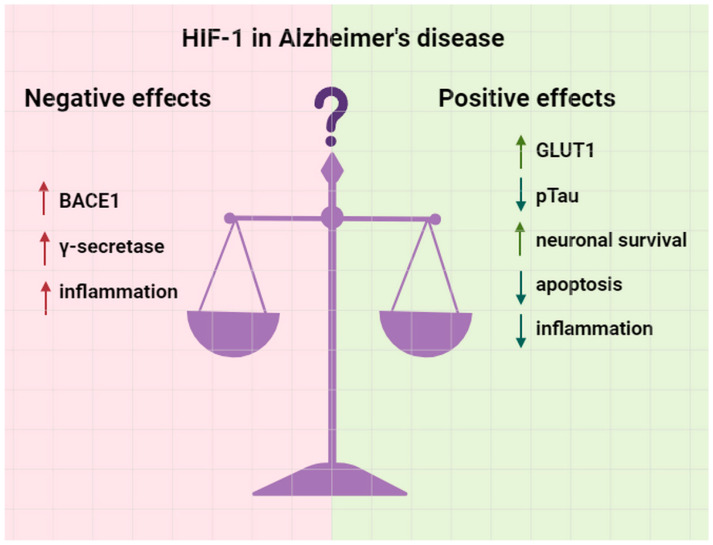
The double-edged sword of HIF-1 in Alzheimer’s disease pathogenesis. Vertical up arrows indicate increasing expression or activity, downward vertical arrows indicate decreased expression or activity. Green arrows indicate positive effects, red arrows indicate negative ones.

Thus, these findings delineate a dual role of HIF-1α in AD pathophysiology (Figure 3).

### 3.3. HIF-1 in Parkinson’s Disease

While the precise molecular mechanisms of PD remain incompletely elucidated, considerable evidence implicates mitochondrial dysfunction and oxidative stress in its pathogenesis [122,123,124]. PD is characterized by mitochondrial deficits, including a reduced mitochondrial number and structural damage, alongside diminished activity of complex I of the mitochondrial respiratory chain, potentially mediated by α-synuclein [125,126,127].

The expression of HIF-1α and its downstream targets is downregulated in the neurons located within the substantia nigra of the brain in individuals with PD [128]. Notably, in a PD mouse model induced by 1-methyl-4-phenyl-1,2,3,6-tetrahydropyridine (MPTP), HIF-1α accumulation was inhibited in dopaminergic PC12 cell lines and mice [129]. 

An essential interplay between HIF-1 and Parkinson’s disease involves tyrosine hydroxylase (TH). TH is responsible for the formation of L-DOPA, a pivotal step in dopamine biosynthesis, thus regulating the rate of the entire biosynthetic process. Parkinson’s disease can be characterized as a syndrome marked by deficient striatal TH levels. Investigations into the activity of the tyrosine hydroxylase gene promoter reveal its regulation by the HRE. Hypoxia and agents that activate HIF-1 have been shown to enhance TH expression in the brain [130]. Additionally, studies suggest that HIF-1α plays a critical role in neuronal and dopaminergic differentiation in vitro. Conditional knockout of HIF-1α in young adult mice led to neuronal alterations and a 40% reduction in TH expression in the substantia nigra [131,132].

Similar to various other neurodegenerative disorders, oxidative stress is a critical factor in the pathogenesis of PD. Dysregulation of ROS balance and the suppression of antioxidant defense mechanisms can lead to lipid peroxidation, protein oxidation, and DNA damage, contributing to the loss of dopaminergic cells in the substantia nigra associated with PD [133]. ROS have been implicated in stabilizing HIF-1α under certain conditions [75]. The mitochondria-targeted antioxidant mitoubiquinone (MitoQ) has been shown to abolish the hypoxia-induced production of ROS and decrease the level of the HIF-1α protein under low-oxygen conditions (3% O_2_), while ROS-generating compounds increase HIF-1α stabilization, even under normoxia [134,135]. Conversely, HIF-1α plays a role in mitigating excessive ROS production by reorganizing mitochondrial respiratory chain complexes’ oxygen consumption and suppressing the expression of mtDNA-encoded mRNAs [75]. The reduction in mitochondrial oxidative respiration and subsequent ROS production observed in PD compared to physiological levels may lead to HIF-1 destabilization.

DJ-1 (PARK7), the loss of which is associated with early PD onset [136], has been shown to negatively regulate the ubiquitination of the von Hippel–Lindau (VHL) α-subunit of HIF-1. DJ-1 modulates HIF-1α stability by influencing the HIF-VHL interaction, and DJ-1 deficiency results in reduced HIF-1α levels in both hypoxia and oxidative stress models. Conversely, elevated levels of HIF-1α and/or its targets have been implicated as a protective mechanism in certain in vitro PD models [137]. The decreased induction of vascular endothelial growth factor (VEGF) mRNA expression downstream of DJ-1 loss suggests the role of DJ-1 in mediating neuronal survival via the VHL-HIF-1α pathway [137].

An increase in iron content specifically within the substantia nigra pars compacta is linked to the biochemical pathology observed in PD. Iron is implicated in oxidative stress by facilitating the release of oxygen free radicals from H_2_O_2_. Studies propose that 6-hydroxydopamine (6-OHDA) induces lesions in the nigrostriatal dopaminergic neurons through metal-catalyzed free radical generation. To investigate this, the impact of the iron chelator deferoxamine (DFO) on 6-OHDA-induced dopaminergic neuron degeneration in rats was examined via intracerebroventricular injection of the inducer. The impact of the iron chelator deferoxamine (DFO), recognized as an inducer of HIF-1, on the 6-OHDA-induced degeneration of the dopaminergic neurons in rats was explored. These studies demonstrated that intracerebroventricular injection of DFO effectively shielded rats from neurodegeneration [138].

**Figure 4:**
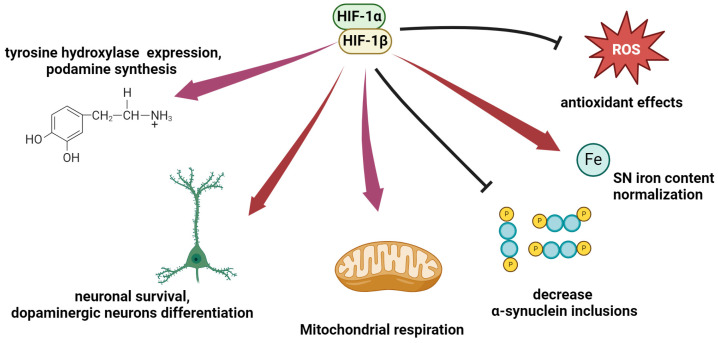
An overview of the potential of HIF-1α stabilization for neuroprotection in Parkinson’s disease. Red and purple arrows indicate strengthening or support. Black lines indicate inhibition of ROS production or α-syn aggregation.

Furthermore, the HIF-1 target genes EPO and VEGF have been identified as contributors to neuronal protection in the context of PD pathogenesis. EPO exhibits neuroprotective effects against the dopaminergic neurotoxin 6-hydroxydopamine, while VEGF offers protection against 1-methyl-4-phenyl-1,2,3,6-tetrahydropyridine (MPTP) toxicity in the substantia nigra cells and may also facilitate dopaminergic neuron growth, proliferation, and differentiation. These findings suggest that HIF-1 may exert a neuroprotective influence on the brain in PD (Figure 4) [131]. This underscores the potential of HIF as a therapeutic target for neurodegenerative diseases.

## 4. Modulation of HIF Activity as a Therapeutic Approach to Neurodegeneration

To modulate HIF activity, inhibitors of HIF hydroxylases are employed. These hydroxylases include prolyl hydroxylases (PHDs) and asparaginyl hydroxylases (FIHs). PHDs facilitate HIF-1α degradation, while FIH inhibits HIF-1 activity [139]. The enzymatic activity of PHDs relies on oxygen, iron, and 2-oxoglutarate [140]. Consequently, PHD activity can be hindered by small molecules either indirectly (noncompetitively), through reducing the cellular levels of oxygen, iron, or 2-oxoglutarate, or directly (competitively) via compounds binding to and obstructing the 2-oxoglutarate-binding site. The inhibition of hydroxylases has shown promise in conferring protective effects in neurodegenerative diseases [141].

Findings from both fundamental research and clinical trials have underscored that the activation of HIF-1 may represent a potent strategy for retarding symptom progression and ameliorating outcomes in AD [142]. For instance, iron chelators have the capacity to activate HIF due to iron serving as one of the cofactors for HIF PHDs. M30, a multitarget iron chelator, has been documented to elevate HIF-1 protein levels and trigger the expression of genes downstream of HIF-1 such as vascular endothelial growth factor and erythropoietin (VEGF and EPO) [104]. Simultaneously, M30 mitigates tau phosphorylation and shields the cortical neurons from amyloid-beta (Aβ) toxicity [143,144,145]. In addition, the M30 prolyl hydroxylase inhibitor, orally administered to mice for 14 days (2.5 mg/kg/day) after the MPTP neurotoxin, significantly increased striatal dopamine levels and increased tyrosine hydroxylase protein levels and activity [146]. These results have been confirmed by other studies [147,148]. Alejandra E Ramirez et al. demonstrated that M30 inhibited α-syn aggregation and decreased the formation of higher-molecular-weight species [149].

Deferoxamine (DFO), a widely utilized HIF-1 inducer, has been investigated in clinical trials involving AD patients and has demonstrated a deceleration of cognitive decline [142,150]. These investigations suggest that augmenting HIF-1 activity holds promise for forestalling neuronal demise and alleviating AD symptoms.

Lactoferrin (Lf) is an iron-binding glycoprotein renowned for its potent iron-chelating abilities, reminiscent of the synthetic chelator deferoxamine (DFO) [151]. These iron-binding properties enable Lf to stabilize the principal adaptive transcription factor, HIF-1α. The administration of Lf has been demonstrated to significantly elevate HIF-1α levels [152]. In murine models, Lf mitigates the dopaminergic neuron damage induced by 1-methyl-4-phenyl-1,2,3,6-tetrahydropyridine (MPTP) and subsequent dyskinesia. Furthermore, the stabilization of HIF-1α by Lf leads to the induction of tyrosine hydroxylase (TH) and several neuroprotective factors, thereby enhancing neuronal viability against MPP+ toxicity in Parkinson’s disease [153]. Lf has also shown promise in ameliorating cognitive impairment in Alzheimer’s disease mouse models through HIF-1α activation [115].

The presence of ferrous ions within the active sites is vital for the activity of prolyl hydroxylase domain-containing enzymes (PHDs). Cobalt can displace the sole free iron in these active sites, thereby deactivating these hydroxylases. Consequently, cobalt has the capacity to stabilize HIF-1α [154,155]. 

Moreover, in addition to cobalt and iron chelators, 2-oxoglutarate analogues have emerged as inhibitors of hydroxylation and inducers of HIF-1α protein. These analogues exhibit greater selectivity toward prolyl hydroxylases (PHDs) and asparaginyl hydroxylases (FIH) than simple iron chelators. Notable examples include 3,4-dihydroxybenzoate (3,4-DHB) and Roxadustat (FG-4592) [156,157]. For instance, 3,4-DHB has shown significant inhibition of FIH, with selectivity over PHDs [157,158]. In one study [159], subacute administration of 1-methyl-4-phenyl-1,2,3,6-tetrahydropyridine (MPTP) at doses of 2 × 20 mg/kg body weight 12 h apart resulted in a 30% loss of dopaminergic neurons in the substantia nigra pars compacta in mice. However, pretreatment with 3,4-DHB for 6 h before the initial administration of MPTP resulted in complete protection against dopaminergic cell loss, as well as preservation of tyrosine hydroxylase levels. Furthermore, DHB pretreatment led to increased levels of HIF-1α and increased expression of HIF-dependent genes such as vascular endothelial growth factor (VEGF) and heme oxygenase-1 (HO-1) [159]. Additionally, DHB demonstrated the ability to prevent microglial activation, resulting in decreased neuronal death both in vitro and in vivo in a PD model. These effects may be related to the ability of DHB to induce an increase in HO-1 levels, thereby eliciting both antioxidant and anti-inflammatory effects [160].

The regular use of iron-chelating HIF-PHDs may potentially lead to adverse effects. such as restless leg syndrome or anemia. Therefore, the exploration of non-iron-targeting HIF-PHDs holds promise in their therapeutic benefits. Roxadustat is an orally administered drug characterized by a short half-life, and when used in conjunction with an intermittent dosing schedule, it induces a transient rise in hypoxia-inducible factor (HIF) activity. It was jointly developed by FibroGen (San Francisco, CA, USA) and Astellas (Tokyo, Japan)/AstraZeneca (Cambridge, UK) and was approved for the treatment of chronic kidney disease in China in December 2018 and in Japan in September 2019 for patients dependent on dialysis [79]. Recent studies have suggested that Roxadustat (FG-4592) may have the ability to partially penetrate the blood–brain barrier and induce the expression of HIF-1α in the brain tissue. Building upon this finding, researchers conducted an experiment on mice where MPTP (30 mg/kg/day) and Roxadustat (10 mg/kg/day) were intraperitoneally administered for 5 consecutive days. The results demonstrated that FG-4592 led to a dose-dependent increase in HIF-1α expression, accompanied by the induction of tyrosine hydroxylase (TH) and the activation of antioxidant enzymes such as Nrf2 and HO-1. Furthermore, a reduction in ROS production was observed, suggesting that these effects of HIF-1α activation may contribute to the protection of dopaminergic neurons against the cytotoxic effects of MPP+. Additionally, FG-4592 ameliorated the behavioral impairments induced by MPTP in mice [161]. However, further research is needed to elucidate the potential impact of FG-4592 on the development of Alzheimer’s disease.

Orexin-A, a neuropeptide produced by the hypothalamic neurons, has garnered attention for its potential neuroprotective properties. Dysfunction in the orexinergic system has been linked to various neurodegenerative disorders, including AD and PD [162,163,164,165]. Orexins play pivotal roles in regulating feeding behavior, energy balance, sleep–wake cycles, autonomic nervous system function, and neuronal survival [166]. Multiple studies have demonstrated that Orexin-A can upregulate the expression of HIF-1 by inhibiting VHL or E3 ubiquitin ligase activity [167,168,169]. In PD pathogenesis, mitochondrial dysfunction may lead to reduced oxygen consumption, thereby activating prolyl hydroxylase and decreasing HIF-1α levels, which subsequently reduces the expression of several transcription factors implicated in protecting the brain against oxidative stress. Elevated levels of HIF-1 protein have been shown to mitigate toxin-induced neuronal damage, particularly by MPP+. Orexin-A not only induces HIF-1α expression but also activates downstream targets such as EPO and VEGF, exerting neuroprotective effects [170].

Furthermore, investigations into the genetic regulation of the HIF-1α gene have yielded promising insights. Chai et al. engineered an rAAV vector containing the human HIF-1α gene (rAAV-HIF-1α), which effectively expressed the HIF-1α protein in primary cultured hippocampal neurons and rat hippocampal tissue [171]. In vitro and in vivo findings suggest that rAAV-mediated HIF-1α gene delivery may ameliorate amyloid-β (Aβ)-induced cognitive deficits by reducing hippocampal neuron apoptosis in Alzheimer’s disease models [171].

Furthermore, augmentation of HIF-1α expression through the utilization of a GV287-based lentiviral vector exhibited improvements in motor dysfunction and pathological alterations in an MPTP-induced PD model. This beneficial outcome was correlated with heightened expression of miR-128-3p, resulting in reduced Axin1 expression and subsequent activation of the Wnt/β-catenin signaling pathway [128].

However, contrasting findings have emerged regarding the neuroprotective role of HIF-1 inhibition. Recent research [172] published in 2024 introduced a novel class of 8-biaryl-2,2-dimethylbenzopyranamide derivatives, with compound D13 exhibiting promising efficacy in animal models associated with Alzheimer’s disease and ischemic stroke. Experimental investigations revealed that compound D13 activates the PI3K/AKT/mTOR signaling pathway while concurrently suppressing HIF-1α and its downstream target genes [172]. In a separate study, knockdown and pharmacological inhibition of HIF-1α during a model of thiamine-deficiency-induced neurotoxicity markedly reduced the formation of BACE1, the C-terminal 99-amino-acid fragment (C99), and the development of AD-like pathology [173].

Moreover, the application of siHIF-1α lentivirus to induce HIF-1 deficiency led to a reduction in hypoxia-induced tau hyperphosphorylation and the restoration of diminished PP2Ac methylation levels. Additionally, this intervention resulted in the amelioration of learning and memory impairments observed in rats subjected to chronic hypoxia [174].

## 5. Conclusions

Hence, given the brain’s heightened energy requirements, it is particularly susceptible to oxygen deprivation, and hypoxic injury can precipitate significant alterations in neuronal function, implicated in neurodegenerative conditions like Alzheimer’s and Parkinson’s disease. Numerous studies propose that modulating HIF-1α activity could mitigate cellular damage in these neurodegenerative disorders by orchestrating the transcription of a diverse array of genes, rendering the stimulation of the HIF-1α-mediated response a promising therapeutic avenue. Nonetheless, apprehension persists regarding the safety of eliciting the full transcriptional response mediated by HIF-1α given the potential for off-target effects. Hence, future research endeavors should prioritize comprehensive drug assessment and the development of safer therapeutic modalities.

The effects of HIF-1 are subject to significant debate. On the one hand, HIF-1 demonstrates the ability to prevent neuronal apoptosis and maintain neuronal differentiation. Additionally, its positive impact may correlate with the upregulation of GLUT1 and GLUT3, facilitating the maintenance of normal brain energy metabolism. Moreover, HIF-1α has been observed to normalize tau protein phosphorylation by activating GLUT1.

Conversely, overexpression of HIF-1α in the brains of individuals with Alzheimer’s disease (AD) has been shown to inhibit the activation of insulin receptor substrate 1 in the neurons. This inhibition leads to insulin resistance and impaired glucose metabolism, contributing to neurodegeneration [175]. 

Additionally, heightened levels of HIF-1 have been associated with inflammation in both the brain and peripheral nervous system, as it directly activates the microglia and astrocytes, fostering neuroinflammation and the progression of various neurodegenerative conditions.

The role of HIF-1 in oxidative stress presents a similarly contentious issue. While HIF-1α can mitigate excessive ROS production by reorganizing oxygen consumption in mitochondrial respiratory chain complexes, several studies have indicated that heightened expression of HIF-1α typically results in increased ROS production by the mitochondria.

The imbalance in the HIF-1-mediated response to hypoxia may represent a novel pathological pathway for neurodegeneration. HIF-1α appears to serve a dual role in AD and PD, functioning as both a neuroprotective agent and a proinflammatory and neurotoxic factor. The specific role of HIF-1 likely depends on the degree and duration of hypoxia, as well as various associated factors.

Thus, in future therapeutic endeavors targeting HIF-1, it will be crucial to focus on maintaining appropriate levels of HIF-1α expression while maximizing its neuroprotective effects and minimizing its proinflammatory and neurotoxic impacts. In the early stages of neurodegeneration and under conditions of mild hypoxia, stimulating HIF-1α expression may decelerate disease progression by reducing inflammation and enhancing neuronal survival. Conversely, in cases of chronic hypoxia, particularly in advanced stages of AD, suppressing HIF-1α expression may prove to be a more efficacious approach.

## List of Abbreviations

3,4-DHB—3,4-dihydroxybenzonate;
6-OHDA—6-hydroxydopamine;
α-syn—α-synuclein.
AAV—adeno-associated virus;
AD—Alzheimer’s disease;
AICD—APP intracellular domain;
ApoE—apolipoprotein E;
APP—amyloid precursor protein;
ARNT—aryl hydrocarbon receptor nuclear translocator—HIF-β subunit;
Aβ—amyloid-β;
BACE-1—beta-site APP-cleaving enzyme 1;
DA—dopamine;
DFO—deferoxamine;
EPO—erythropoietin;
FIH—FIH—asparaginyl hydroxylase;
GLUT 1—glucose transporter-1;
GLUT 3—glucose transporter-3;
HIF—hypoxia-inducible factor;
HO-1—heme oxygenase-1;
HRE—hypoxia-responsive elements, short DNA sequences within the gene promoter;
IH—intermittent hypoxia;
LDHA—lactate dehydrogenase A;
Lf—lactoferrin;
MPP+—1-methyl-4-phenylpyridine;
MPTP—1-methyl-4-phenyl-1,2,3,6-tetrahydropyridine;
NFT—neurofibrillary tangles;
ODDD—oxygen-dependent degradation domain;
O-GlcNAc—O-linked N-acetylglucosamine;
OSA—obstructive sleep apnea;
PD—Parkinson’s disease;
PDK1—pyruvate dehydrogenase 1;
PHD—prolyl hydroxylase;
pVHL—von Hippel–Lindau protein;
ROS—reactive oxygen species;
sAPPβ—soluble APPβ;
SNc—compact part of the substantia nigra;
TH—tyrosine hydroxylase;
VEGFA—vascular endothelial growth factor A
